# RaspberryJalisco: A field-collected multi-class dataset of Raspberry (*Rubus idaeus*) phenological stages from Mexican commercial orchards with YOLOv8 benchmarks

**DOI:** 10.1016/j.dib.2026.113253

**Published:** 2026-09-16

**Authors:** Jaime Adan Cuevas-Ramirez, Carlos Alberto Villaseñor-Padilla, Javier Gomez-Avila, Carlos Lopez-Franco, Jesus Hernandez-Barragan, Gehova Lopez-Gonzalez, Nancy Arana-Daniel

**Affiliations:** Division of Technologies for Cyber-Human Integration (DTIC), Centro Universitario de Ciencias Exactas e Ingenierias (CUCEI), Universidad de Guadalajara, Blvd. Marcelino Garcia Barragan 1421, Guadalajara, Jalisco, 44430, Mexico

**Keywords:** Object detection, Precision agriculture, Annotated images, Bounding boxes, Fruit counting, Yield estimation, Phenological staging, Machine learning

## Abstract

We present RaspberryJalisco, a field-collected annotated image dataset of raspberry (*Rubus idaeus*) fruits at distinct phenological stages, captured in a commercial orchard in Jocotepec, Jalisco, Mexico, during November–December 2025. The dataset comprises 11,884 tiled images and 174,763 bounding-box annotations distributed across two complementary labeling schemes: a fine-grained 5-class configuration (Bud, BrightRed C4, DarkRed C5, Green, Orange (red dot)) and a coarser 3-class configuration (Immature, BrightRed C4, Orange (red dot)) designed for yield-prediction and harvest-planning tasks. Images were acquired using a DSLR camera under natural midday illumination and manually annotated by three domain-expert annotators with LabelImg. In response to the risk of parent-image leakage after tiling, the revised release includes persistent parent-image identifiers, random and parent-image grouped split manifests, a leakage audit, corrected random-split baselines, and leakage-resistant grouped YOLOv8 benchmarks. All annotations are provided in YOLO normalized format, with dataset descriptors, scripts, source tables, figures, and YOLOv8 benchmark weights. The data can be reused for in-domain fruit detection, fruit counting, phenological analysis, model benchmarking, and method development, while cross-site or cross-season deployment should be validated externally.

Specifications Table

**Table:** 

Subject	Agricultural and Food Engineering; Computer Vision and Pattern Recognition
Specific subject area	Multi-stage phenological annotation and object-detection benchmarking of raspberry (Rubus idaeus) fruits under real orchard conditions
Type of data	Images (.jpg); YOLO annotation files (.txt); dataset descriptors (data.yaml); YOLOv8 model weights (.pt); Python training and analysis scripts
Data collection	Images were acquired with a DSLR camera under natural midday illumination in a commercial raspberry orchard in Jocotepec, Jalisco, Mexico (November-December 2025). Bounding boxes were manually annotated using LabelImg [1] by three domain-expert annotators and curated through Roboflow [2].
Data source location	Jocotepec, Jalisco, Mexico (Lake Chapala region; approx. 20 deg 17 min N, 103 deg 26 min W).
Data accessibility	Repository name: Zenodo. Data identification number: 10.5281/zenodo.20766567. Direct URL to data: https://doi.org/10.5281/zenodo.20766567. Instructions for accessing these data: The dataset is publicly accessible without restriction. The revision package includes random and grouped split manifests, corrected benchmark tables, per-class metrics, stratified size/occlusion metrics, confusion matrices, figures, source tables, data YAML files, and reproducibility commands.
Related research article	None.

## 1 Value of the Data

• **First Mexican raspberry orchard dataset.** To the best of our knowledge, this is the first publicly available annotated image dataset of raspberry fruits collected in Mexican commercial orchards. Existing benchmarks such as RaspberrySet [3] were collected under European growing conditions; the present dataset was acquired under the Lake Chapala micro-climate, characterized by dense tropical foliage, high relative humidity, variable canopy shadows, and asynchronous within-plant ripening.
• **Dual class-configuration design.** Two complementary annotation schemes are provided. The 5-class configuration supports fine-grained phenological analysis (bud, ripe, over-ripe, unripe, transitional), while the 3-class configuration aggregates pre-harvest stages into a single Immature class, supporting yield-prediction and harvest-planning tasks.
• **Leakage-audited YOLOv8 benchmarks.** YOLOv8 models (nano through extra-large) were evaluated under random and parent-image grouped split protocols for both class configurations, offering researchers accuracy reference values under the original tile-level split and a stricter leakage-resistant protocol. Training scripts, hyperparameters, model weights, source tables, and reproducibility commands are included.
• **Large-scale, densely annotated collection.** With 174,763 bounding-box annotations across 11,884 images (mean 14.7 instances/image; median 11), the dataset reflects the high annotation density typical of raspberry canopies. >87% of annotations belong to the Immature class.
• **High-resolution tiled acquisition.** Original DSLR images were captured at 4000 × 3000 px, downsampled to 2000 × 1500 px, and subsequently tiled into non-overlapping 640 × 640 px sub-images, a strategy commonly used for small-object detection in dense canopy environments [4].
• **Scope-limited reuse.** The dataset can support training object detectors for fruit counting and phenotyping, benchmarking model scale, studying class imbalance, and developing methods for dense small-fruit canopies. Results should be interpreted as in-domain references for the Lake Chapala acquisition setting; transfer to other orchards, seasons, cultivars, cameras, or illumination regimes requires external validation.

## 2 Background

Raspberry (*Rubus idaeus*) is a high-value soft fruit with growing global demand; Mexico is a leading exporter, with Jalisco - particularly the Lake Chapala region - accounting for approximately 40% of national production [5]. Manual inspection is impractical given labor shortages, dense canopy occlusion, and the asynchronous ripening of buds, green, transitional, and ripe fruits on the same cane, motivating single-stage detectors such as the YOLO family [6,7] for real-time fruit detection [8]. RaspberrySet [3] established an early reference point; prior work by the present authors reached mAP@50 = 0.740 with YOLOv8m [9], with limitations in minority-class detection and occlusion handling.

Because the final images are tiles from higher-resolution photographs, tile-level random splitting can place related tiles from the same parent photograph across subsets, inflating apparent generalization. We report both the original random split and a revised parent-image grouped split assigning all tiles from one source photograph to a single subset.

Recent multi-domain evidence shows that strong in-domain mAP does not guarantee field robustness under domain shifts such as illumination, occlusion, cultivar morphology, or acquisition site [10]. We therefore interpret the present benchmarks as controlled in-domain, leakage-audited reference values requiring external validation before wider deployment.

## 3 Data Description

### 3.1 Repository structure

Upon downloading and extracting the dataset archive [11], the directory is organized as follows:

**Table:** 

RaspberryJalisco/
|–– data_5classes.yaml
|–– data_3classes.yaml
|–– train/
| |–– images/ (8318 .jpg files)
| `–– labels/ (8318 .txt files)
|–– valid/
| |–– images/ (2378 .jpg files)
| `–– labels/ (2378 .txt files)
`–– test/
|–– images/ (1188 .jpg files)
`–– labels/ (1188 .txt files)

Each .txt label file follows the YOLO normalized annotation format: <class_id> x_c y_c w h, where $x_{c}$, $y_{c}$, w, and h∈[0,1] are normalized with respect to image width and height.

### 3.2 Dataset statistics

Table 1 summarizes images and instances per split. The original dataset was partitioned using Roboflow’s automated random split with a 70/20/10 train/validation/test ratio. The revised supplementary package also includes parent-image grouped split manifests to support leakage-resistant evaluation.

**Table 1:** Dataset split statistics. Images include both annotated frames and background (empty) frames.

Split	Images	Instances	Mean inst./image	Median inst./image
Train	8318	122,085	14.7	11
Validation	2378	34,961	14.7	11
Test	1188	17,717	14.9	11
Total	11,884	174,763	14.7	11

### 3.3 Split manifests and leakage audit

Each tiled image is associated with a persistent parent_image_id derived from the source photograph identifier and tile offsets. The revision package includes two split manifests: grouped_split_manifest.csv and random_split_manifest.csv. Each manifest contains tile_id, parent_image_id, x_offset, y_offset, split, image_filename, and label_filename. The grouped split assigns all tiles from a parent image to a single subset, while the random split records the original tile-level random partition used as the historical baseline.

The leakage audit quantified parent-image overlap between subsets. In the random split, 1529 parent images were shared between train and validation, 927 between train and test, and 607 between validation and test. In the grouped split, all pairwise parent-image overlaps were zero. The grouped split therefore satisfies the revised leakage-resistant evaluation criterion.

### 3.4 Class definitions and distribution

5-class configuration (Fig. 2).

1. **Bud** (class 0): compact, rounded floral bud; green to pale-yellow colouration.
2. **BrightRed C4** (class 1): fully ripe fruit; commercially harvestable stage.
3. **DarkRed C5** (class 2): over-ripe or senescent fruit; darker, less glossy red.
4. **Green** (class 3): unripe green drupelets; dominant class by instance count.
5. **Orange (red dot)** (class 4): transitional stage; orange drupelets with a visible central red dot.

3-class configuration (Fig. 3).

1. **Immature** (class 0): aggregation of Bud, Green, and DarkRed C5.
2. **BrightRed C4** (class 1): as above.
3. **Orange (red dot)** (class 2): as above.

**Fig. 3:**
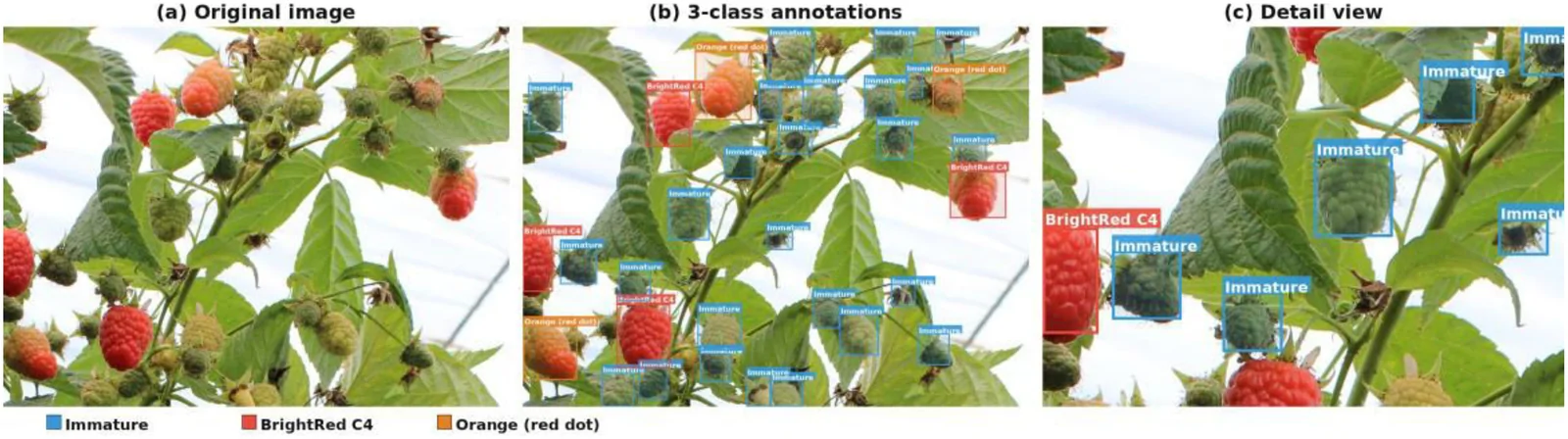
Representative sample image from the dataset under the 3-class configuration. (a) Original DSLR image (2000×1500 px tile). (b) Ground-truth bounding-box annotations under the 3-class configuration: Immature, BrightRed C4, Orange (red dot). (c) Detail view of a high-density canopy region illustrating the co-occurrence of multiple phenological stages on a single cane.

The class counts in Table 2 are visualized in Fig. 4.

**Table 2:** Per-class instance distribution across splits (3-class configuration).

Class	Train	Validation	Test	Total	%
Immature	107,130	30,654	15,515	153,299	87.7%
BrightRed C4	10,335	3036	1485	14,856	8.5%
Orange (red dot)	4620	1271	717	6608	3.8%
Total	122,085	34,961	17,717	174,763	100.0%

**Fig. 4:**
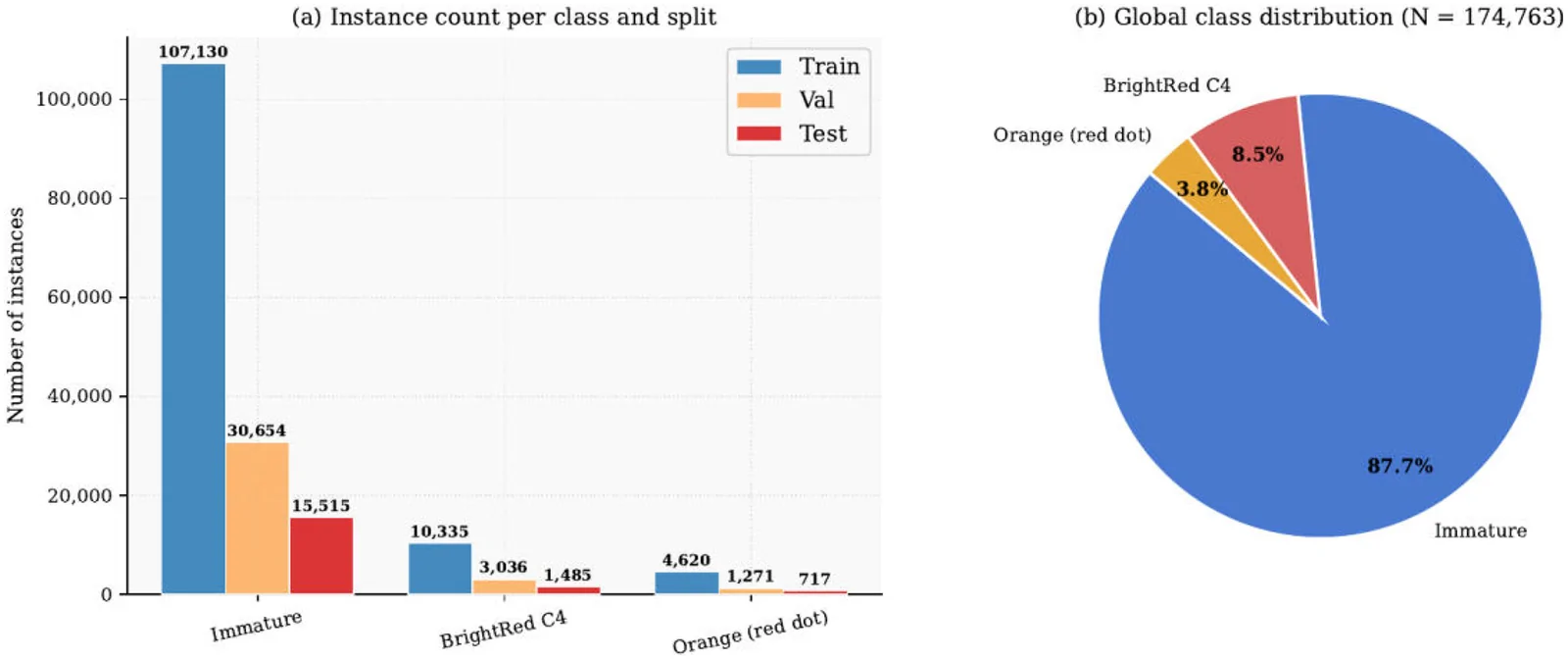
Class distribution across dataset splits. (a) Instance counts per class and split. (b) Global proportions (3-class configuration; N = 174,763).

### 3.5 Bounding-box geometry

Table 3 reports bounding-box statistics. Boxes are small (mean normalized area ≈ 0.0051, equivalent to roughly 32 px × 42 px at 640 × 640), consistent with dense canopy structure and high-resolution tiling.

**Table 3:** Bounding-box geometry statistics across all 174,763 annotations (YOLO normalized coordinates, values in [0,1]). P99: 99th percentile.

Metric	Mean	Std	Min	Q1	Median	Q3	P99
Width	0.0554	0.0314	0.0009	0.0335	0.0474	0.0691	0.1617
Height	0.0729	0.0427	0.0002	0.0436	0.0621	0.0913	0.2171
Area	0.0051	0.0072	0.0000	0.0015	0.0029	0.0061	0.0316
Aspect ratio	0.8225	0.3748	0.0822	0.6522	0.7500	0.9000	2.5385

### 3.6 Spatial distribution and annotation density

The spatial distribution of normalized bounding-box center coordinates across all 174,763 annotations is near-uniform, indicating that the tiling strategy preserved annotation coverage across the full image area. The number of annotated instances per image has mean = 14.7, median = 11, P95 = 41, and maximum = 113.

## 4 Experimental Design, Materials and Methods

### 4.1 Study site and image acquisition

Images were collected in a commercial raspberry orchard in the municipality of Jocotepec, Jalisco, Mexico (Lake Chapala region). Image acquisition was conducted during November–December 2025, covering successive harvest cycles. All images were acquired under natural midday illumination (10:00–14:00 h local time) using a DSLR camera at a native resolution of 4000 × 3000 px, hand-held at approximately 30–60 cm from the plant row with a slightly lateral angle.

### 4.2 Image preprocessing and tiling

Raw images at 2000 × 1500 px were partitioned into non-overlapping tiles of 640 × 640 px using a sliding-window approach, yielding the 11,884 tiles that constitute the training corpus. This strategy preserves fine-grained texture and color information used to discriminate between spectrally similar classes [4].

### 4.3 Manual annotation

Bounding boxes were drawn using LabelImg [1] (v1.8.6) by a team of three domain-expert annotators. Each annotator worked independently on a designated subset; ambiguous cases were resolved by consensus. A senior annotator performed a final quality-control pass over 100% of annotations. The dataset was then imported into Roboflow [2] for the original random split, export to YOLOv8 format, and generation of both class variants. No image-level augmentation was applied in Roboflow; all augmentation was performed on-the-fly by the Ultralytics training engine.

### 4.4 Grouped split and stratified evaluation protocol

The grouped split was reconstructed from tile-level metadata by grouping records by parent_image_id before assigning them to train, validation, and test. Split integrity was assessed by computing the set intersection of parent_image_id values for train-validation, train-test, and validation-test pairs. A grouped split was considered leakage-free only when all three intersections were zero.

To avoid ambiguity between class schemes, the revised package uses immutable data views for random five-class, random three-class, grouped five-class, and grouped three-class labels. The random five-class view contains 8318 train, 2378 validation, and 1188 test images with class IDs 0–4. The random three-class view preserves the same image membership but stores remapped class IDs 0–2.

Fruit-size stratification was computed from bounding-box area after scaling normalized YOLO box width and height to a 640 × 640 px reference frame. Small, medium, and large bins were defined by the one-third and two-third area quantiles within each class configuration. Occlusion was approximated by the maximum IoU between a ground-truth object and any neighboring ground-truth object in the same tile. Objects with maximum neighbor IoU below 0.10 were assigned to low occlusion, objects from 0.10 to below 0.30 to medium occlusion, and objects at or above 0.30 to high occlusion.

### 4.5 YOLOv8 benchmark experiments

#### 4.5.1 Model family

YOLOv8 [7] is a single-stage anchor-free detector built on CSPDarknet53 with a decoupled detection head and composite loss:

$$L=\lambda_{\text{box}}\;L_{\text{box}}+\lambda_{\text{cls}}\;L_{\text{cls}}+\lambda_{\text{dfl}}\;L_{\text{dfl}}$$

with $\lambda_{\text{box}}=7.5$, $\lambda_{\text{cls}}=0.5$, $\lambda_{\text{dfl}}=1.5$ [7]. $L_{\text{box}}$ uses CIoU [12]; $L_{\text{cls}}$ is binary cross-entropy; $L_{\text{dfl}}$ is Distribution Focal Loss. Five scale variants were evaluated: nano (n), small (s), medium (m), large (l), and extra-large (x).

The composite loss includes CIoU bounding-box regression [12], binary cross-entropy classification loss, and Distribution Focal Loss. Revision experiments used Ultralytics 8.4.104 and PyTorch 2.8.0 on a CPU server (Intel Xeon Platinum 8592 V). Performance was assessed on the held-out test split using Precision (P), Recall (R), mAP@50, and mAP@50–95 (Table 4).

**Table 4:** Fixed training hyperparameters for all YOLOv8 experiments.

Hyperparameter	Value
Optimizer	SGD
Initial LR (lr0)	0.008
Final LR (lrf)	0.01
Momentum	0.937
Weight decay	0.0005
Epochs	100
Batch size	64
Image size	640 × 640
Warmup epochs	3
Early stopping patience	10
AMP	Disabled
*Online augmentation*	
Mosaic	0.15
Copy-paste	0.05
Horizontal flip	0.5
Rotation (±)	10${}^{\circ }$
Translation	0.05
Scale	0.15

Corrected YOLOv8 test results under random and parent-image grouped split protocols (Table 5). The corrected random five-class baseline was revalidated on restored five-class labels.

**Table:** **Table 5**.

#### 4.5.2 Evaluation metrics

Performance was assessed on the held-out test split using Precision (P), Recall (R), mAP@50, and mAP@50–95 (standard COCO metric). Per-class precision, recall, AP@50, and AP@50–95 were extracted from the Ultralytics validation API. Numeric confusion matrices were computed with confidence threshold 0.25 and IoU threshold 0.45, then normalized by true-class row. Stratified AP and recall were computed with detections matching same-class ground truth outside the target stratum ignored for that stratum.

## 5 Results

The leakage audit showed substantial parent-image overlap in the original random split but none in the grouped split (Fig. 1). After correcting the random five-class labels, five-class random-vs-grouped differences were small: mAP@50 differences ranged from −0.017 to 0.003, where negative values indicate grouped outperforming random. In contrast, the three-class random split was consistently higher than grouped by 0.002 to 0.032 mAP@50, with the largest gaps for YOLOv8l and YOLOv8x. These results indicate that parent-image leakage can affect performance estimates, but the magnitude and direction of the effect depend on class configuration and model scale (Fig. 5).

**Fig. 1:**
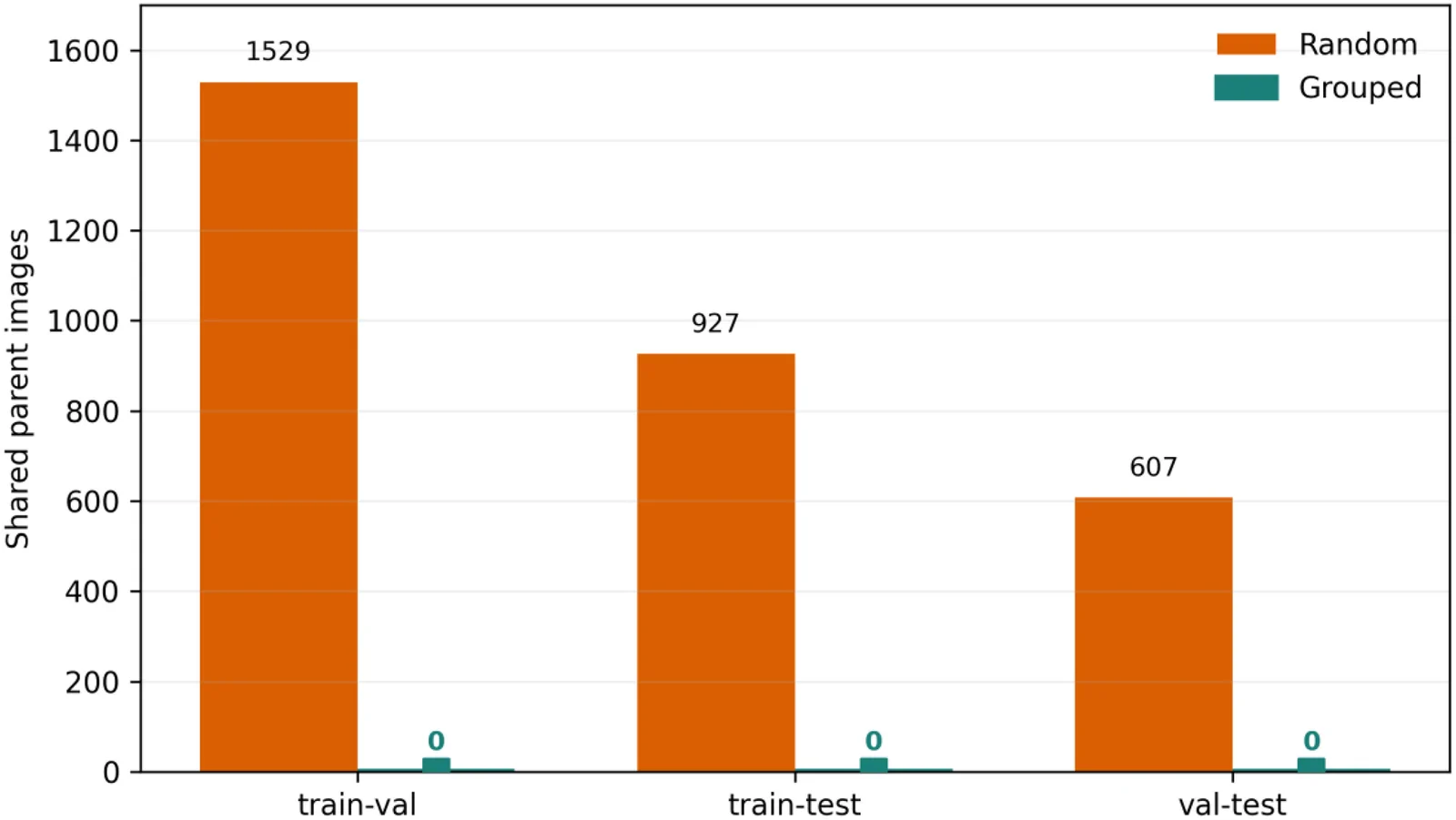
Parent-image overlap audit for the original tile-level random split and the revised parent-image grouped split. The grouped split eliminates parent-image overlap across all train/validation/test pairs.

**Fig. 2:**
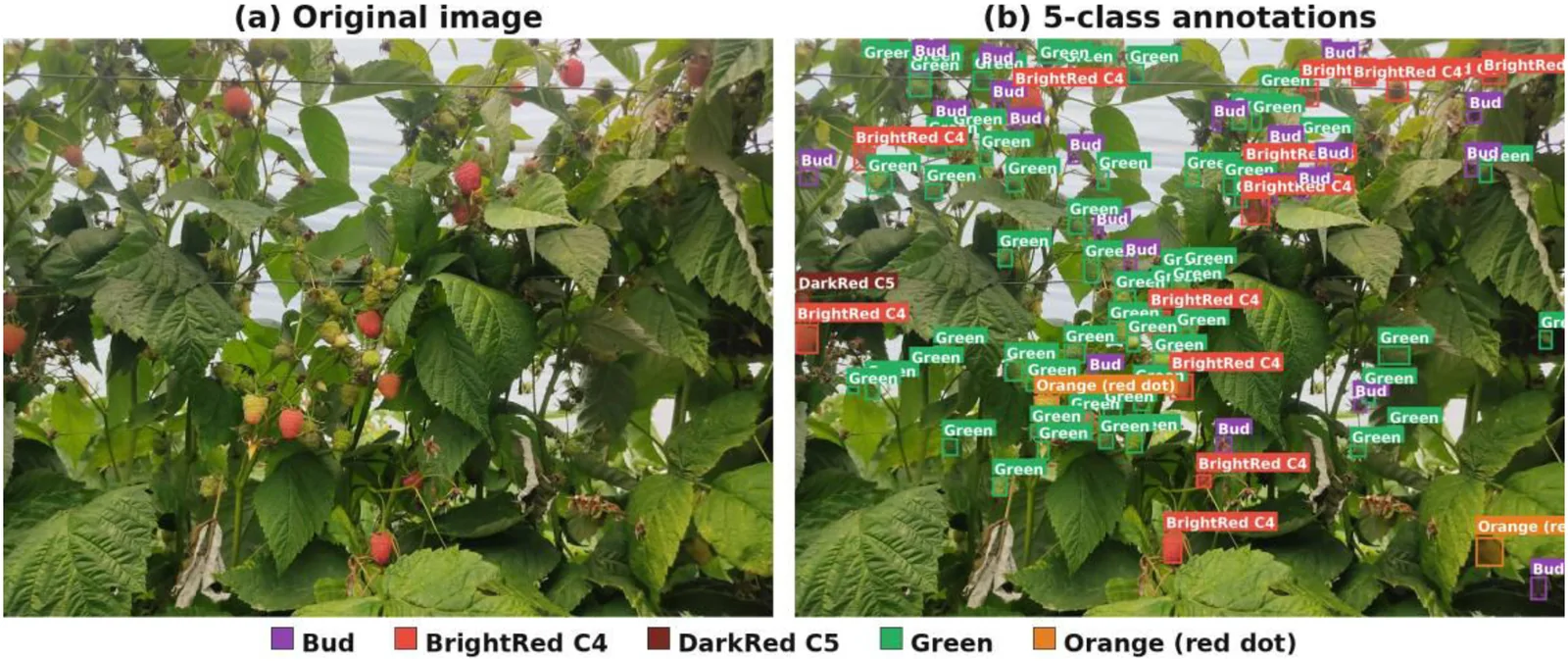
Representative sample image with ground-truth bounding-box annotations under the 5-class configuration.(a) Original image (2000×1500 px tile). (b) Annotated image: Bud, BrightRed C4, DarkRed C5, Green, Orange (red dot).

**Fig. 5:**
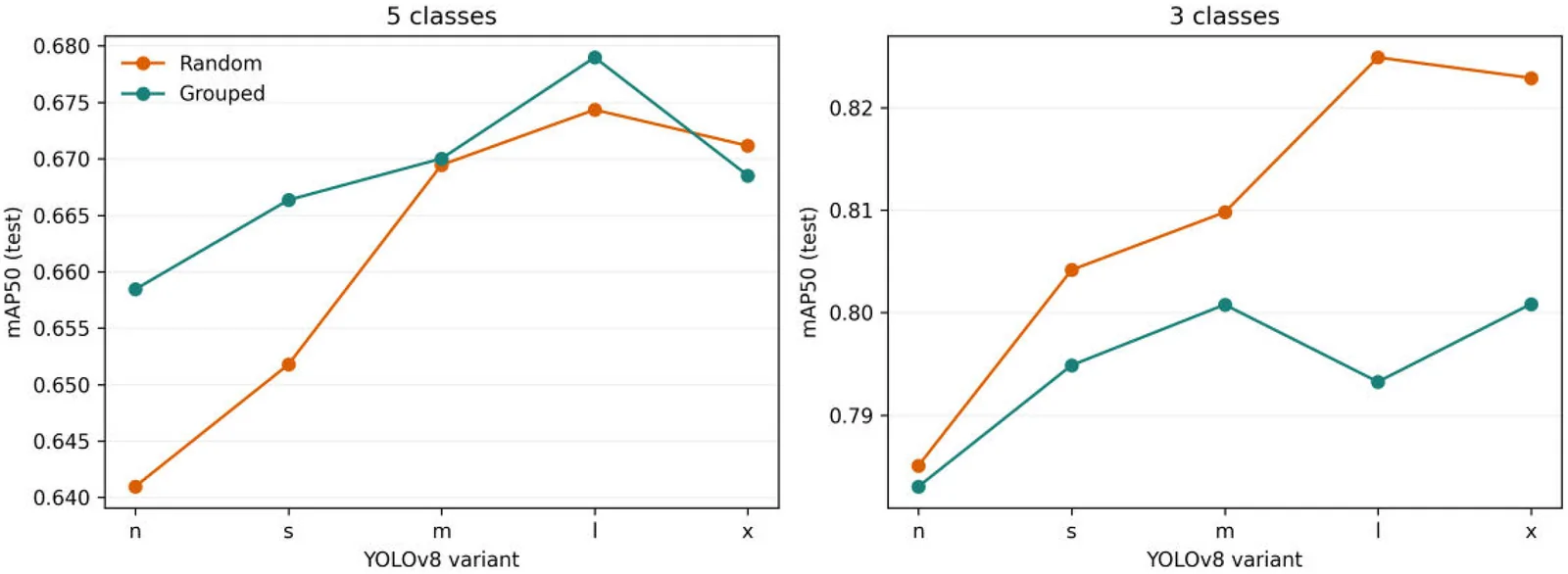
Corrected YOLOv8 mAP@50 comparison under random and parent-image grouped split protocols for five-class and three-class configurations.

For the grouped five-class protocol, YOLOv8l obtained the highest mAP@50–95 (P = 0.627, R = 0.657, mAP@50=0.679, mAP@50–95=0.338). For the grouped three-class protocol, YOLOv8x obtained the highest mAP@50–95 (P = 0.766, R = 0.743, mAP@50=0.801, mAP@50–95=0.425), with YOLOv8m close behind (mAP@50=0.801, mAP@50–95=0.424).

For the best grouped five-class model (YOLOv8l), BrightRed C4 and Green were the strongest classes, with AP@50–95 of 0.462 and 0.413, respectively. Orange (red dot) also remained robust (AP@50–95=0.411). Bud and DarkRed C5 were substantially harder, with AP@50–95 of 0.200 and 0.204, respectively; DarkRed C5 also had only 130 test instances in the grouped test split. For the best grouped three-class model (YOLOv8x), BrightRed C4 achieved the highest AP@50–95 (0.462), followed by Orange (red dot) (0.423) and Immature (0.389) (Fig. 6, Fig. 7).

**Fig. 6:**
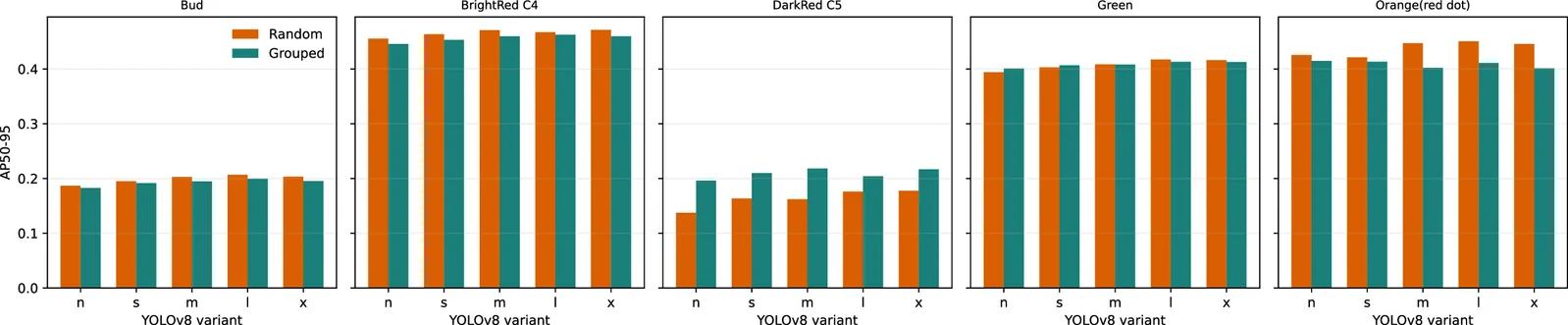
Per-class AP@50–95 for corrected five-class random and grouped split evaluations across YOLOv8 model scales.

**Fig. 7:**
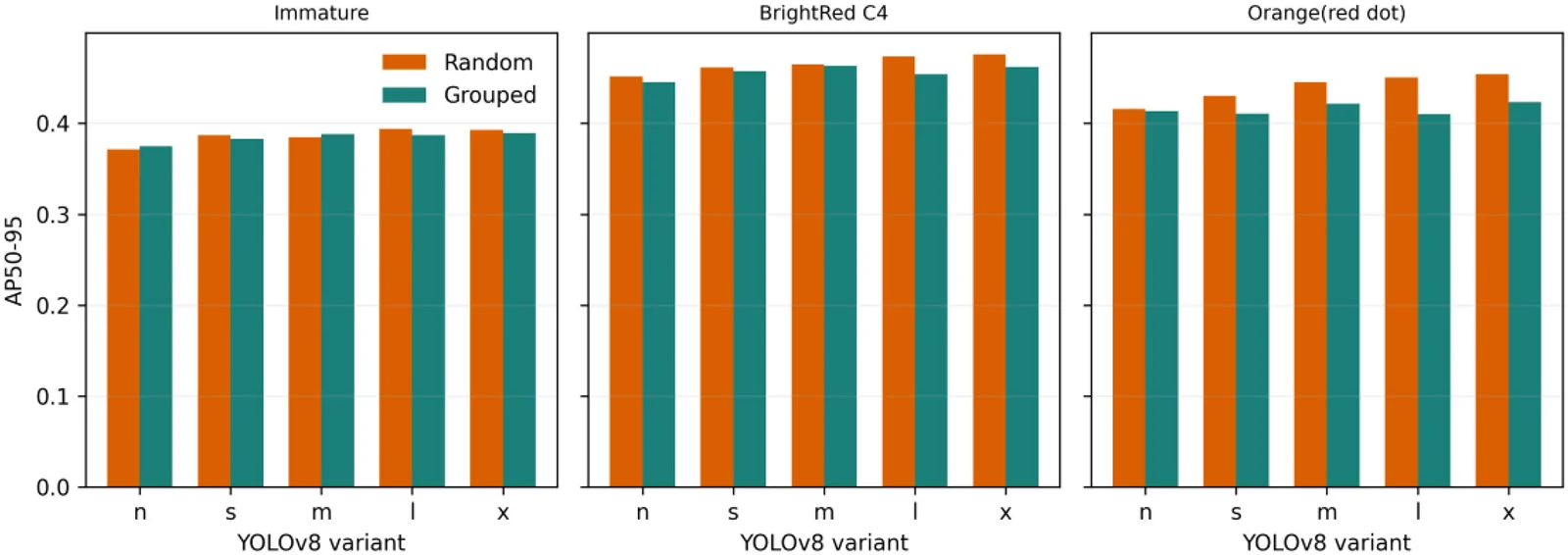
Per-class AP@50–95 for three-class random and grouped split evaluations across YOLOv8 model scales.

Performance increased with fruit size. For the best grouped five-class model, AP@50–95 increased from 0.076 for small objects to 0.222 for medium objects and 0.271 for large objects. For the best grouped three-class model, AP@50–95 increased from 0.078 for small objects to 0.254 for medium objects and 0.361 for large objects. Occlusion produced an even stronger performance gradient. For the best grouped five-class model, AP@50–95 declined from 0.285 under low occlusion to 0.085 under medium occlusion and 0.004 under high occlusion. For the best grouped three-class model, AP@50–95 declined from 0.339 under low occlusion to 0.144 under medium occlusion and 0.007 under high occlusion (Fig. 8, Fig. 9). Class-wise confusion patterns for the best-performing grouped models are shown in Fig. 10.

**Fig. 8:**
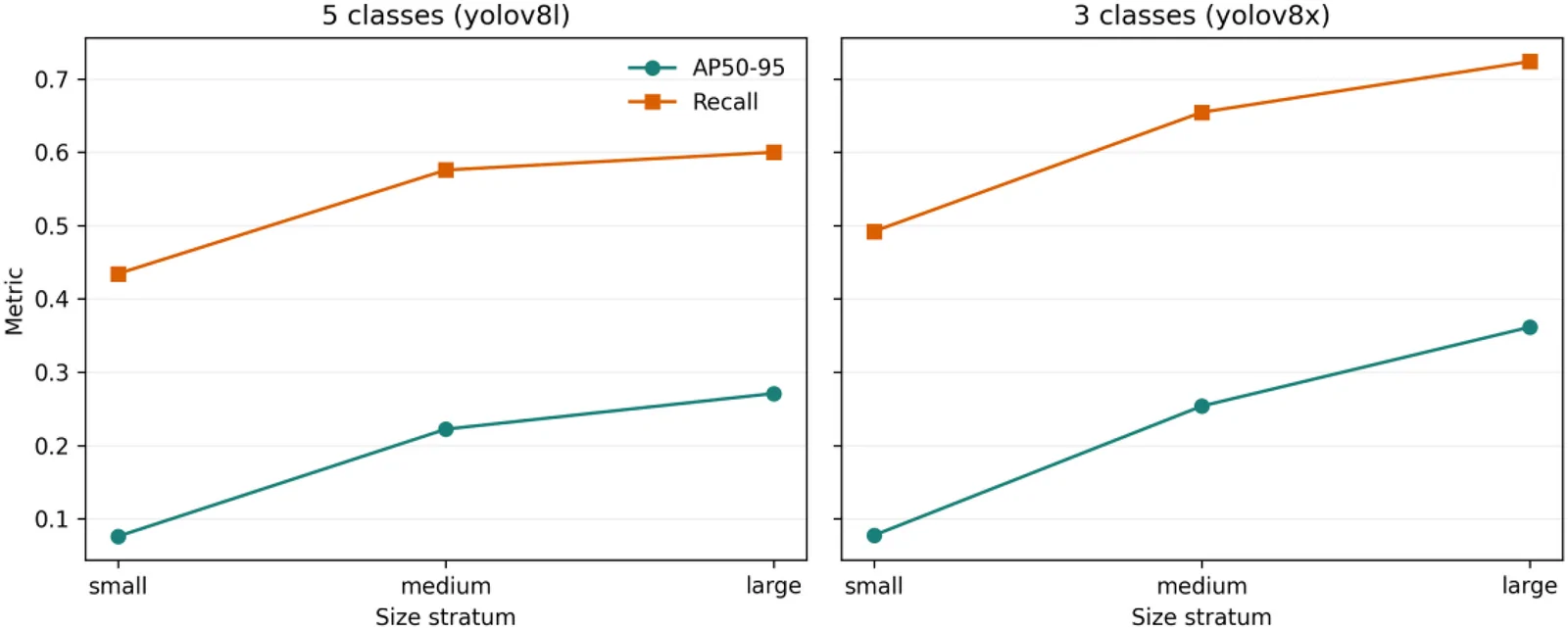
Size-stratified AP@50–95 and recall for the best grouped five-class and three-class models. Small, medium, and large bins are quantile-based using bounding-box area at a 640×640 px reference scale.

**Fig. 9:**
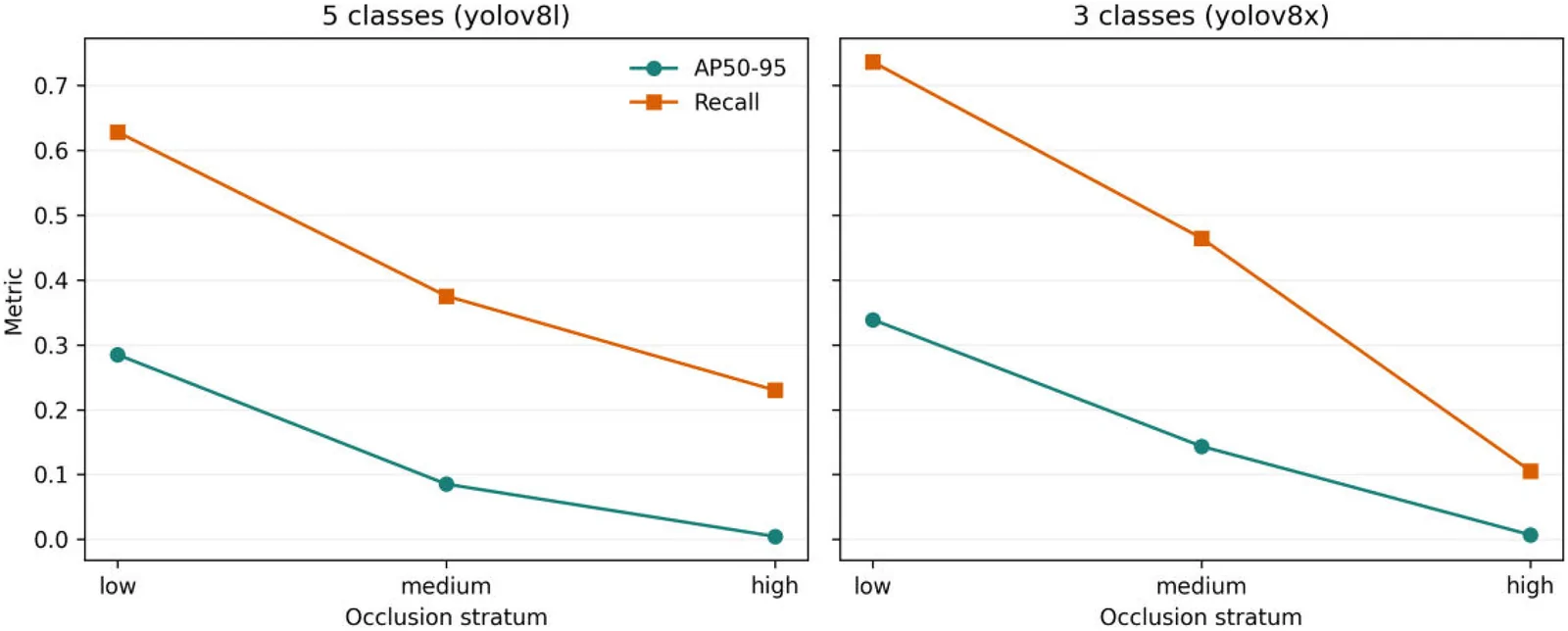
Occlusion-stratified AP@50–95 and recall for the best grouped five-class and three-class models. Occlusion is approximated by maximum IoU with neighboring ground-truth boxes in the same tile.

**Fig. 10:**
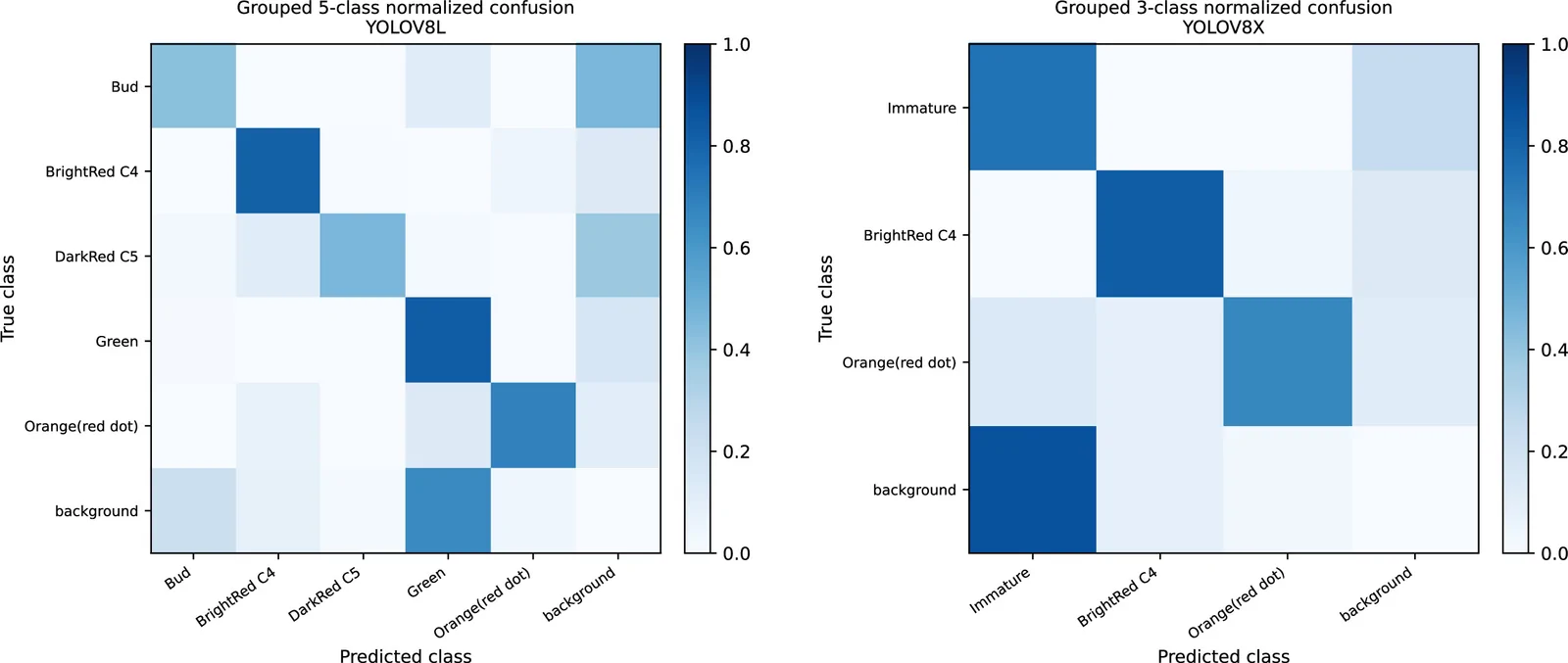
Grouped normalized confusion matrices for the best grouped 5-class model (YOLOv8l, left) and best grouped 3-class model (YOLOv8x, right).

Training convergence curves, baseline (non-grouped) confusion matrices, precision-recall and F1-score curves, qualitative detection examples, and representative annotated training batches are provided for reference in Fig. 11, Fig. 12, Fig. 13, Fig. 14, Fig. 15.

**Fig. 11:**
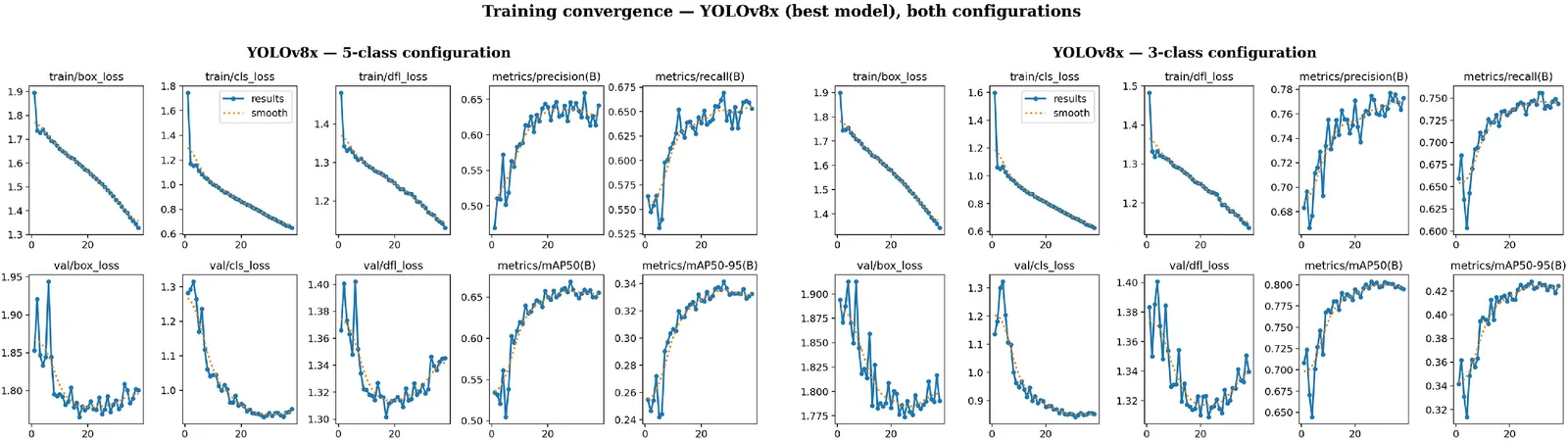
Training convergence curves for YOLOv8x under the 5-class and 3-class configurations.

**Fig. 12:**
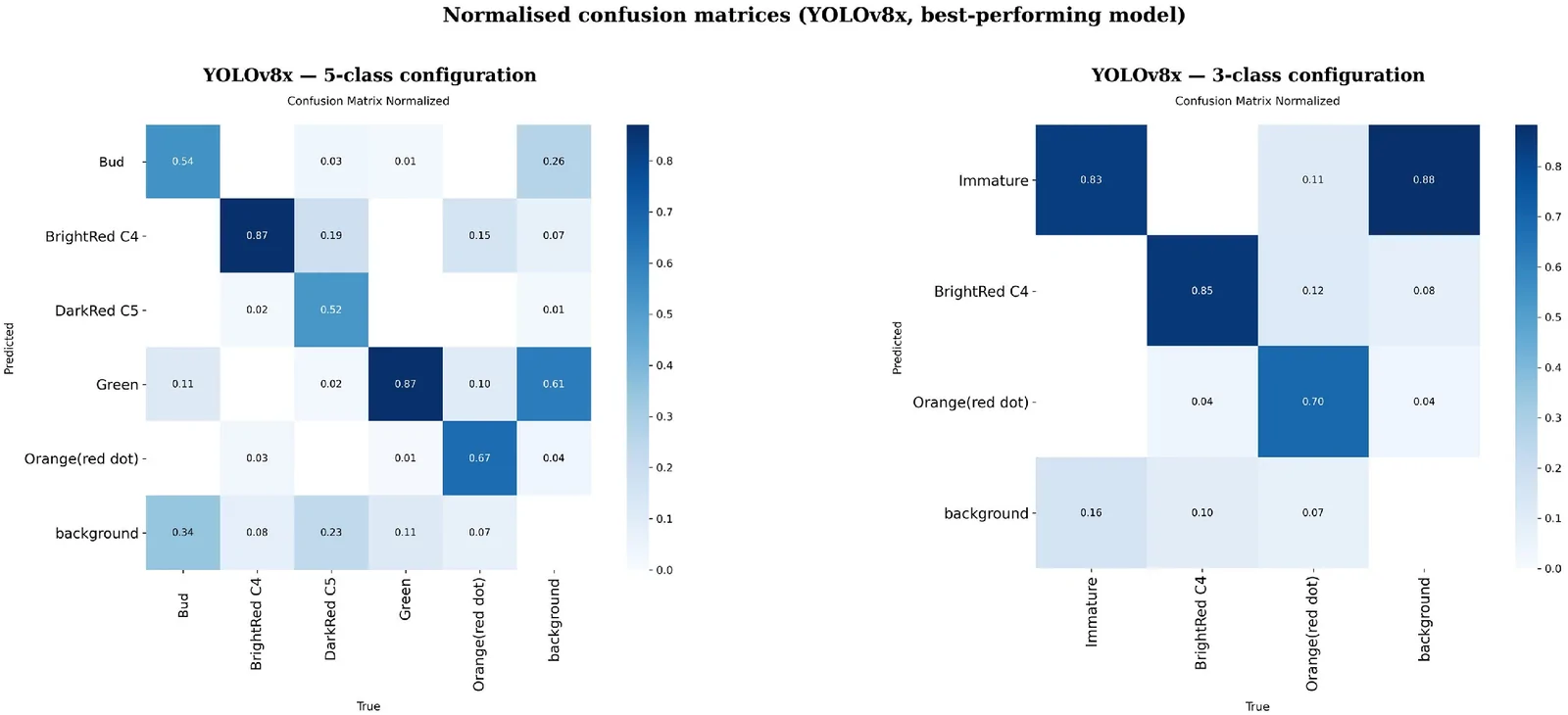
Baseline YOLOv8x normalized confusion matrices on the original test split, retained from the initial benchmark for reference.

**Fig. 13:**
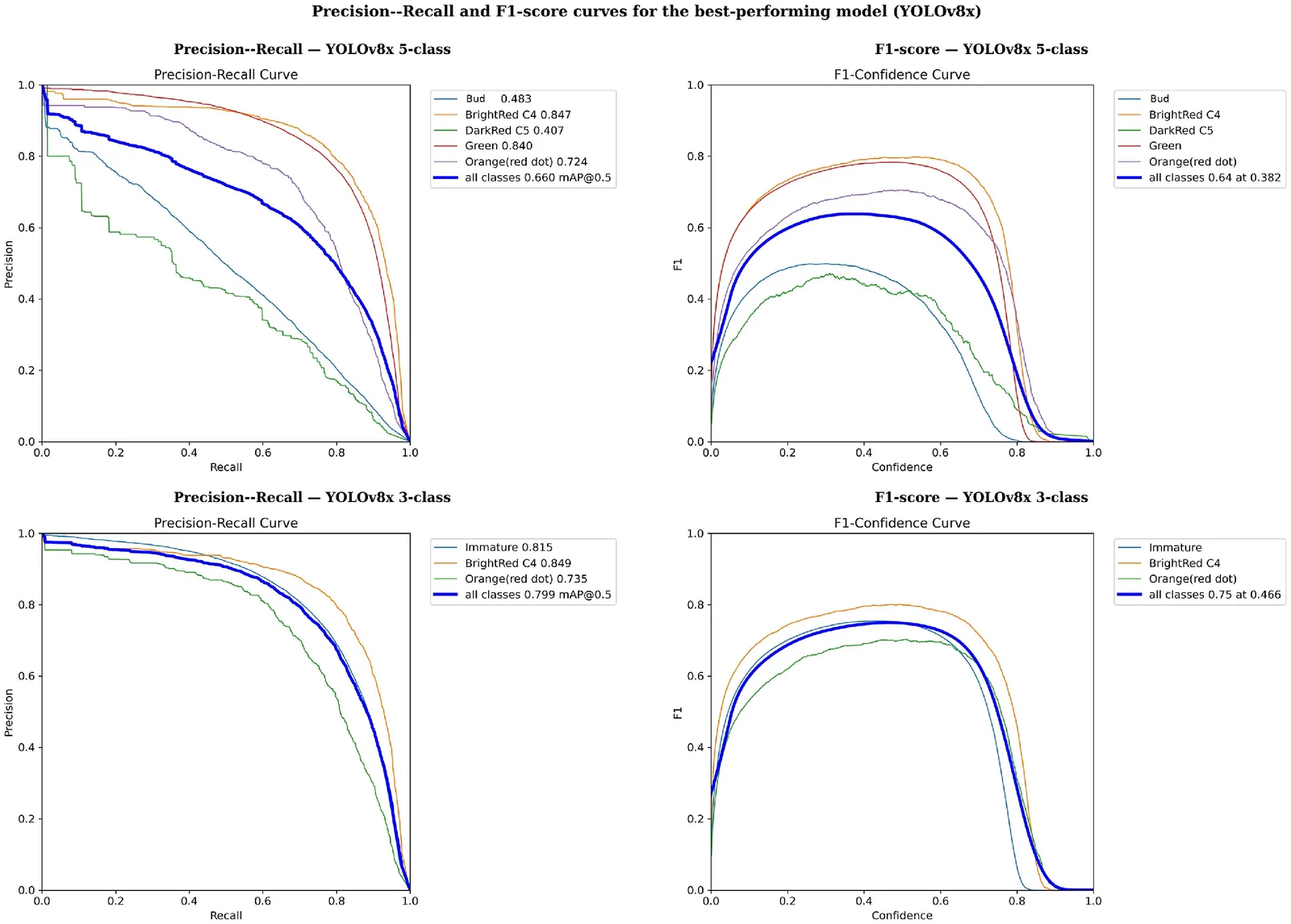
Precision–Recall and F1-score curves for YOLOv8x.

**Fig. 14:**
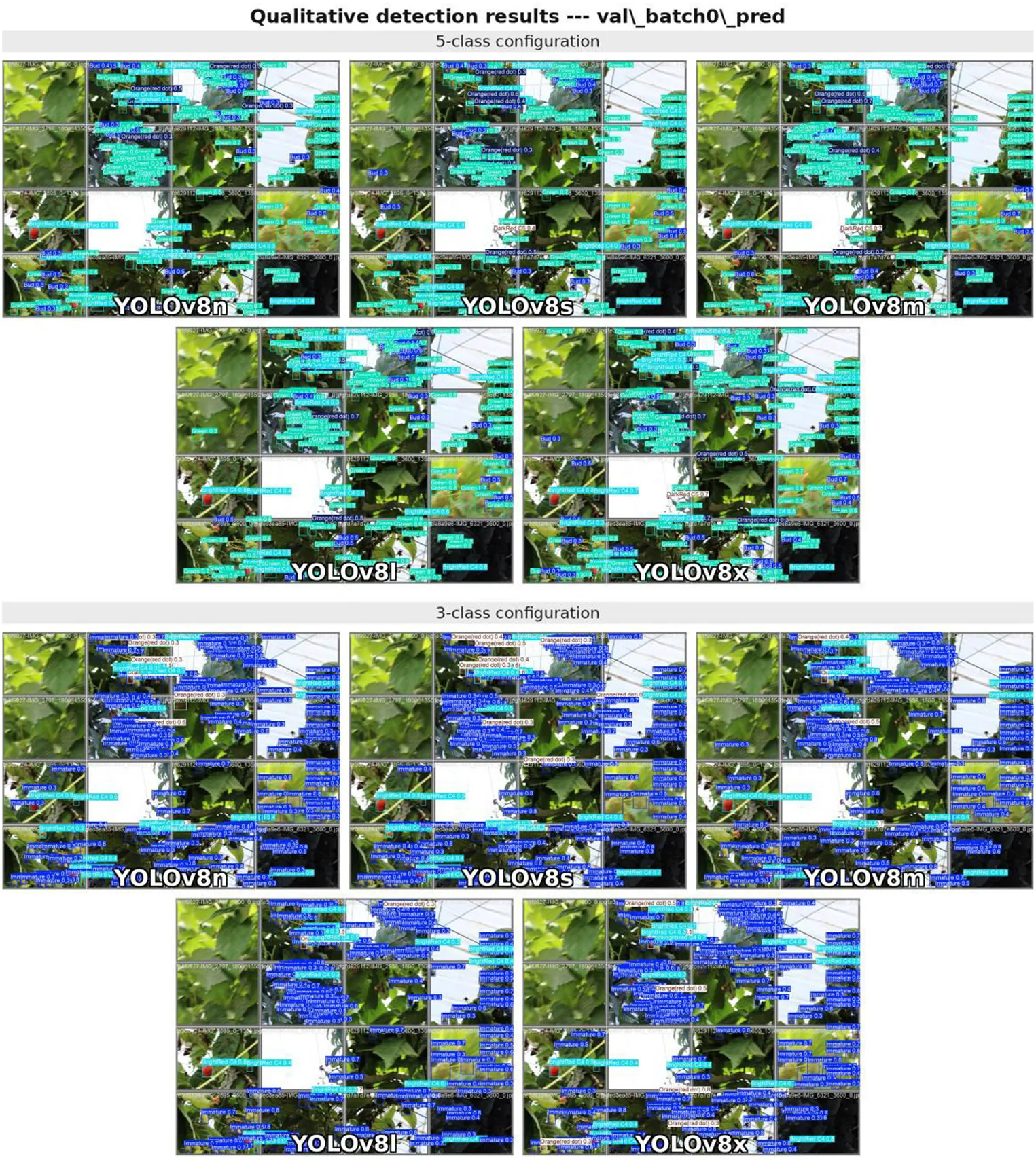
Qualitative detection results on a representative validation batch for all ten YOLOv8 models.

**Fig. 15:**
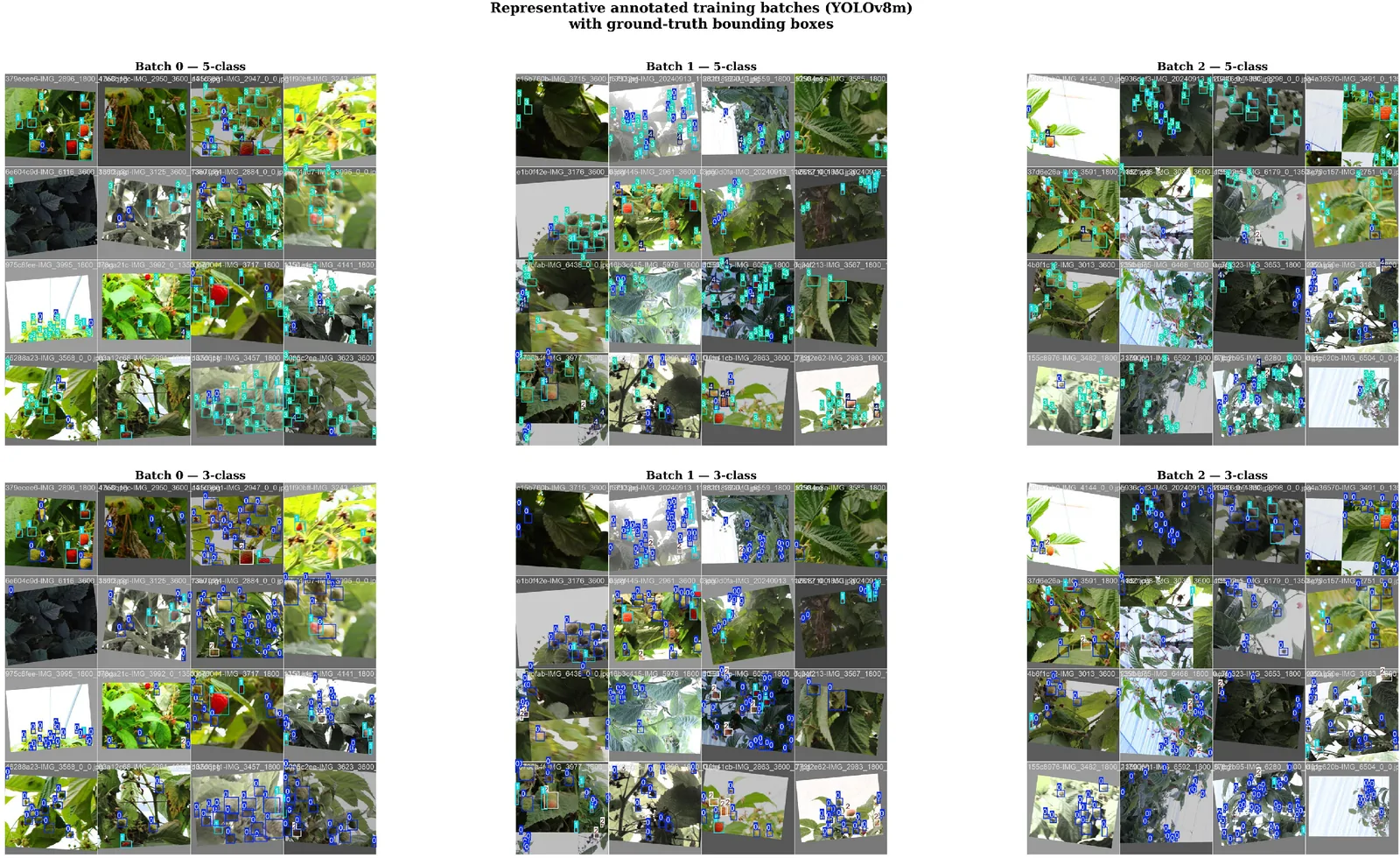
Representative annotated training batches with ground-truth bounding boxes.

## Limitations

Several limitations should be considered. First, all images were collected at a single geographic site (Jocotepec, Jalisco), limiting generalization to other climatic zones. Second, data cover a single seasonal window; intra-season phenological transitions are not represented. Third, although the revised grouped split removes parent-image leakage among train, validation, and test subsets, it does not substitute for external validation on independent orchards, cultivars, seasons, or acquisition hardware. Fourth, dense canopy occlusion and small fruit size remain important failure modes, as shown by the stratified AP analysis. Fifth, annotations are bounding-box only; segmentation masks are not included. Sixth, benchmark inference times were measured on CPU only and may differ on GPU or edge-AI accelerators.

## Ethics Statement

The authors confirm that this work does not involve human subjects, animal experimentation, or data collected from social media platforms. All images were captured on privately owned agricultural land with the explicit permission of the farm owners. No identifiable human subjects are present in the dataset.

## CRediT Author Statement

**Jaime Adan Cuevas-Ramirez:** Data curation, Formal analysis, Investigation, Methodology, Software, Visualization, Writing – original draft. **Nancy Arana-Daniel:** Funding acquisition, Conceptualization, Project administration, Supervision, Writing – review & editing. **Carlos Alberto Villaseñor-Padilla:** Supervision, Validation, Writing – review & editing. **Javier Gomez-Avila:** Validation, Writing – review & editing. **Carlos Lopez-Franco:** Supervision, Writing – review & editing. **Jesus Hernandez-Barragan:** Validation, Writing – review & editing. **Gehova Lopez-Gonzalez:** Software, Writing – review & editing.

## Data Availability

ZenodoRaspberryJalisco: A multi-class dataset on the phenological stages of Rubus idaeus with YOLOv8 annotations (Original data).

## Appendix Supplementary materials

Image, application 1
